# Prevalence of antibiotic resistance genes in drinking and environmental water sources of the Kathmandu Valley, Nepal

**DOI:** 10.3389/fmicb.2022.894014

**Published:** 2022-08-22

**Authors:** Mohan Amarasiri, Tsubasa Takezawa, Bikash Malla, Takashi Furukawa, Jeevan B. Sherchand, Eiji Haramoto, Kazunari Sei

**Affiliations:** ^1^Laboratory of Environmental Hygiene, School of Allied Health Sciences, Kitasato University, Sagamihara, Japan; ^2^Interdisciplinary Center for River Basin Environment, University of Yamanashi, Kofu, Japan; ^3^Institute of Medicine, Tribhuvan University Teaching Hospital, Kathmandu, Nepal

**Keywords:** antibiotic resistance genes, aquatic environments, ddPCR, drinking water, Kathmandu Valley

## Abstract

Antibiotic-resistant bacteria-associated infections are responsible for more than 1.2 million annual deaths worldwide. In low- and middle-income countries (LMICs), the consumption of antibiotics for human and veterinary uses is not regulated effectively. Overused and misused antibiotics can end up in aquatic environments, which may act as a conduit for antibiotic resistance dissemination. However, data on the prevalence of antibiotic resistance determinants in aquatic environments are still limited for LMICs. In this study, we evaluated the prevalence and concentration of antibiotic resistance genes (ARGs) in different drinking and environmental water sources collected from the Kathmandu Valley, Nepal, using droplet digital polymerase chain reaction to understand the current situation of ARG contamination. River water and shallow dug well water sources were the most contaminated with ARGs. Almost all samples contained *sul1* (94%), and *intI1* and *tet*(A) were detected in 83 and 60% of the samples, respectively. Maximum ARG concentration varied between 4.2 log_10_ copies/100 ml for *mecA* and 9.3 log_10_ copies/100 ml for *sul1*. Significant positive correlations were found between ARGs (*r* > 0.5, *p* < 0.01), except for *mecA, qnrS*, and *vanA*. As *sul1* and *intI1* were detected in almost all samples, the presence of these genes in a given sample may need to be considered as background antibiotic resistance in LMICs. Therefore, monitoring of ARGs, such as β-lactam ARGs, quinolone resistance genes, and vancomycin resistance genes, may provide a better picture of the antibiotic resistance determinants in aquatic environments of LMICs.

## Introduction

Antibiotic resistance has been identified as a major health threat of the 21^st^ century by the World Health Organization. According to a recent systematic analysis conducted using data from 204 countries, an estimated 4.95 million deaths were associated with antimicrobial resistance (AMR) in 2019, while 1.27 million deaths were attributed to AMR. South Asia had the highest number of deaths associated with and attributed to AMR (1.39 million and 389,000, respectively) (Murray et al., 2022).

Low- and middle-income countries (LMICs) have to overcome significant challenges to control antibiotic resistance dissemination. Human and animal antibiotic consumption is not regulated effectively in many LMICs, and antibiotics, antibiotic-resistant bacteria (ARB), and antibiotic resistance genes (ARGs) surveillance programs are scarce. Fraction of the antibiotics administered to humans and livestock is excreted in the active form and will end up in wastewater (Kümmerer, 2009). Around 80% of the wastewater generated in the world is discharged into aquatic environments without any treatment (United Nations World Water Assessment Programme, 2017). Moreover, available wastewater treatment infrastructure that can remove these contaminants is limited or not well maintained in LMICs, exacerbating the problem (Laxminarayan et al., 2020; Larsson and Flach, 2021). These wastewater streams containing antibiotics, ARB, and ARGs can contaminate the aquatic environments and contribute to the dissemination of antibiotic resistance (Amarasiri et al., 2020). A recent analysis highlighted the importance of improving basic sanitation in reducing global antibiotic resistance (Graham et al., 2019). Therefore, studies on the antibiotics, ARB, and antibiotic resistance gene (ARG) contamination of aquatic environments in LMICs are needed to be conducted as a first step to understand the extent of the problem and developing mitigation measures.

Earlier studies have highlighted many instances of unnecessary antibiotic prescriptions in Nepal, mostly by unqualified medical professionals (Walson et al., 2001; Acharya and Wilson, 2019). Bacterial strains conferring resistance to commonly available and cheaper antibiotics, such as tetracycline and β-lactam antibiotics, are detected even in rural areas without any healthcare facilities, while the resistant strains to antibiotics that are new, costly, and harder to obtain were found mainly in urban areas with many healthcare facilities (Walson et al., 2001). A recent review on the ARB in the human health sector of Nepal has reported high percentages of resistant strains to antibiotics, including cotrimoxazole, ciprofloxacin, ciprofloxacin, tetracyclin, erythromycin, and nalidixic acid (Acharya and Wilson, 2019). *sul1, tet*(B), *bla*_*CTX–M*_, *bla*_*NDM*–1_, and *qnrS* genes were detected in hospital effluents, wastewater treatment plant effluents, and river water, emphasizing the impact of untreated and improperly treated wastewater in contaminating aquatic environments (Thakali et al., 2021). *bla*_*TEM*_, *ermF, mecA*, and *tet*(A) genes were detected from the middle and downstream samples of the Bagmati River flowing through the Kathmandu Valley, confirming the anthropogenic contribution (Thakali et al., 2020). To date, there is only one study on the presence of ARGs in drinking water sources utilized in the Kathmandu Valley (Thakali et al., 2022). Therefore, in this study, we evaluated the ARG concentrations in drinking water sources (surface water and groundwater) and environmental waters in the Kathmandu Valley using the droplet digital polymerase chain reaction (ddPCR) method. *sul1, tet*(A), *tet*(M), *bla*_*KPC*_, *bla*_*OXA*_, *mecA, qnrA, qnrS*, and *intI1* genes were selected based on previous studies reporting their presence in other aquatic samples (Thakali et al., 2020, 2021). A number of hospitals and animal health-related studies have reported the high prevalence of bacterial strains resistant to cotrimoxazole, vancomycin, and aminoglycosides (Nepal et al., 2012; Acharya and Wilson, 2019; Central Veterinary Laboratory, 2021; Pandey et al., 2021). *acrD* and *dfr13* genes were selected to represent the aminoglycoside and cotrimoxazole resistance genes (Adrian et al., 2000; Grape et al., 2005; Szczepanowski et al., 2009). Quaternary ammonium compounds resistance gene, *qacF*, which was used in evaluating the anthropogenic contamination of water sources, was also quantified (Szekeres et al., 2018).

## Methodology

### Study site description

In this study, water samples were collected from the Kathmandu Valley. Kathmandu, the capital city of Nepal, with an area of 664 km^2^ and a population of 2.51 million (Central Bureau of Statistics, 2012), is situated in the central hilly region of Nepal and mainly consists of three major land covers, namely, built-up, agriculture/open space, and forest/shrub (Uddin et al., 2015; Regmi et al., 2017). The municipal piped water in the valley is supplied by Kathmandu Upatyaka Khanepani Limited (2022) that distributes treated surface and groundwater. However, there is a huge supply deficit of piped water in the valley (Kathmandu Upatyaka Khanepani Limited, 2022). Because of this, people in the valley are compelled to use alternative water sources, such as shallow dug and tube wells, deep tube wells, stone spouts, jar water, and tanker water, for drinking and domestic purposes (Shrestha et al., 2017a). However, previous studies have reported the detection of pathogens, such as *Cryptosporidium, Giardia*, human adenoviruses, noroviruses (Haramoto et al., 2011), and *Acinetobacter* spp. (Ghaju Shrestha et al., 2017), as well as human and animal fecal contamination in these water sources (Malla et al., 2015, 2018a,b, 2019). Similarly, river water, which is extensively used for irrigation of vegetable farms in the valley, is highly contaminated with pathogens (Shrestha et al., 2017b), and the interconnection between river water and groundwater of the Kathmandu Valley (Bajracharya et al., 2020) indicates a high possibility of pathogen contamination of groundwater.

### Sample collection, bacterial enumeration, and deoxyribonucleic acid extraction

In total, 36 water samples were collected from deep tube wells (*n* = 6), rivers (*n* = 6), shallow dug wells (*n* = 11), shallow tube wells (*n* = 3), water springs (*n* = 4), and stone spouts (*n* = 6) which are used for drinking and other domestic purposes, between February and September 2016 (Supplementary Figure 1). Total coliforms and *Escherichia coli* were quantified using Colilert reagents (IDEXX Laboratories, Westbrook, CA, United States). Briefly, one pack of Colilert powder was added to 103 ml of a serially diluted water sample, shaken gently until dissolved, and poured into a Quanti-Tray 2000 and sealed. Subsequently, the tray was incubated at 37°C for 24 h. After incubation, yellow-colored large and small wells were observed which were counted as total coliforms, whereas blue-fluorescent large and small wells when observed under UV light were counted as *E*. *coli*. Finally, the result was interpreted as a most probable number (MPN) value using an MPN generating software (IDEXX Laboratories), considering the dilution ratios.

For river water samples, 10 ml was filtered using a disposable filter unit (diameter, 47 mm; pore size, 0.22 μm; Nalgene, Tokyo, Japan), while for other sample types, 100 ml was filtered. Deoxyribonucleic acid (DNA) extraction from the membrane filters was done using the CicaGeneus DNA Extraction Kit (Kanto Chemical, Tokyo, Japan) (Malla et al., 2018b; Ghaju Shrestha et al., 2019). Extracted DNA was stored at −25°C until quantification.

### Droplet-digital polymerase chain reaction for antibiotic resistance gene quantification

Genes conferring resistance to commonly used antibiotics in this region were selected for the analysis (Thakali et al., 2020, 2021). ddPCR was employed in quantifying the ARGs from the drinking water sources and environmental samples speculating that ARG concentrations in drinking water sources may be low, and quantitative PCR (qPCR) may provide below the detection limit results. Primer pairs used to analyze the ARGs are shown in Supplementary Table 1 (Eurofins Genomics, Tokyo, Japan) (Demarta et al., 1999; Turner et al., 1999; Fang and Hedin, 2003; Guillard et al., 2011; Monteiro et al., 2012; Sandberg et al., 2018). gBlocks (Integrated DNA Technologies, Coralville, IA, United States) were used as positive controls, and their sequences are also shown in Supplementary Table 1. ARG concentrations in the samples were quantified using QX200 AutoDG droplet digital PCR system (Bio-Rad, Hercules, CA, United States). All the reagents were purchased from Bio-Rad, Hercules, CA, United States. The reaction mixture contained 11 μl of 2 × ddPCR EvaGreen Supermix, 0.3 μl each of forward and reverse primers (10 μM concentration), 1.1 μl of DNA template, and 9.3 μl of PCR-grade water. Prepared reaction mixtures were loaded to a ddPCR 96-well plate and sealed with a PCR plate heat seal foil using a PX1 PCR plate sealer. The sealed plate was inserted into the automated droplet generator, and droplets were generated in a new ddPCR 96-well plate. The sample plate was sealed as before, and a PCR was conducted with the following cycling conditions on generated droplets using a C1000 Touch thermal cycler. Enzyme activation at 95°C for 5 min was followed by 40 cycles of denaturation at 95°C for 30 s and annealing at 58°C for 1 min. After that, two signal stabilization steps of 5 min were conducted at 4 and 90°C. After the PCR, samples were allowed to cool at 4°C for 15 min and then transferred to the QX200 droplet reader (Bio-Rad). Data acquisition and analysis were done using QX Manager Software (version 1.1) (Bio-Rad). All the reactions resulted in more than 10,000 droplets in this study. Two positive and three negative control samples were included in each run. The threshold of each analysis was determined manually for each plate based on the positive and negative control sample data (Hughes et al., 2022). Each sample was analyzed in triplicate and considered positive if at least 2 wells were positive.

### Data analysis

Absolute ARG concentrations in each sample were expressed as log_10_ gene copies/100 ml. The relative abundance of ARGs was calculated based on the 16S rRNA concentration of each sample. Correlation analysis between ARGs, *E. coli*, and total coliforms was conducted using *corrplot* package in R (Wei and Simko, 2021). Non-metric multidimensional scaling (NMDS) analysis was conducted using *vegan* and *ggord* packages to evaluate dissimilarities in ARG concentrations depending on the source water type (Oksanen et al., 2020; Beck, 2021). All the figures, except for the location map, were developed using the *ggplot2* package using RStudio (Version 4.1.2) (Wickham, 2016; R Core Team, 2021).

## Results

The presence and concentration of total coliforms and *E. coli* in all samples were confirmed by the Colilert method (Malla et al., 2018b; Ghaju Shrestha et al., 2019). All samples contained total coliforms ranging between 1.21 and 7.76 log_10_ MPN/100 ml, and a river water sample contained the highest total coliform concentration (Supplementary Figure 2). *E. coli* was present in all samples with concentrations ranging between −1.00 and 7.38 log_10_ MPN/100 ml. Similar to total coliforms, a river water sample contained the highest *E. coli* concentration (Supplementary Figure 2). All samples were positive for the 16S rRNA gene, and a river water sample had the highest concentration (10.73 log_10_ gene copies/100 ml) (Table 1).

**TABLE 1:** Concentration of each antibiotic resistance gene (ARG) and *intI1* in different drinking and environmental water samples (log_10_ copies/100 ml) (mean ± SD).

Sample name	Source type	*16S*	*sul1*	*tet*(A)	*acrD*	*qacF*	*bla*_*OXA*_	*qnrS*	*tet*(M)	*bla*_*KPC*_	*dfr13*	*qnrA*	*vanA*	*mecA*	*intI1*	Total ARGs
KTM358	DTW	8.04 ± 0.02	4.61 ± 0.22	0	0	0	4.24 ± 0.12	0	0	0	0	0	0	0	4.21 ± 0.02	2
KTM361		8.12 ± 0.04	5.71 ± 0.08	5.44 ± 0.08	4.28 ± 0.20	0	0	0	0	0	0	0	0	0	5.34 ± 0.06	3
KTM436		7.76 ± 0.02	4.55 ± 0.38	4.27 ± 0.17	0	0	0	0	0	0	0	0	0	0	4.58 ± 0.35	2
KTM441		6.28 ± 0.02	4.49 ± 0.30	4.34 ± 0.24	0	0	0	0	0	4.18 ± 0.01	0	0	0	0	4.18 ± 0.00	3
KTM442		7.09 ± 0.02	4.38 ± 0.18	0	0	0	0	4.52 ± 0.23	0	0	0	0	0	0	4.20 ± 0.01	2
KTM463		7.73 ± 0.04	5.45 ± 0.14	4.18 ± 0.01	0	0	0	0	0	0	0	0	0	0	6.04 ± 0.04	2
KTM289	River	7.98 ± 0.09	6.26 ± 0.25	5.69 ± 0.44	0	0	0	0	0	5.17 ± 0.01	0	0	0	0	5.64 ± 0.39	3
KTM298		10.50 ± 0.01	9.34 ± 0.07	8.62 ± 0.04	7.88 ± 0.01	7.83 ± 0.03	7.00 ± 0.03	8.43 ± 0.04	7.98 ± 0.02	7.43 ± 0.03	6.53 ± 0.15	5.68 ± 0.48	6.23 ± 0.15	0	9.14 ± 0.03	11
KTM316		10.46 ± 0.02	9.18 ± 0.06	8.33 ± 0.02	7.64 ± 0.12	7.72 ± 0.02	6.20 ± 0.28	7.95 ± 0.03	7.49 ± 0.05	5.57 ± 0.41	6.26 ± 0.05	5.82 ± 0.15	0	0	8.87 ± 0.01	10
KTM346		10.40 ± 0.04	9.06 ± 0.03	8.34 ± 0.02	7.69 ± 0.03	7.72 ± 0.04	6.50 ± 0.10	8.21 ± 0.05	7.34 ± 0.02	6.70 ± 0.02	6.26 ± 0.09	5.77 ± 0.11	0	0	8.92 ± 0.02	10
KTM373		10.73 ± 0.02	9.15 ± 0.02	8.43 ± 0.02	7.69 ± 0.02	7.55 ± 0.02	6.03 ± 0.15	7.99 ± 0.05	7.63 ± 0.06	6.99 ± 0.04	5.86 ± 0.16	5.59 ± 0.17	5.19 ± 0.02	0	9.03 ± 0.02	11
KTM374		8.79 ± 0.03	6.74 ± 0.08	6.01 ± 0.12	0	0	0	0	0	0	0	0	5.23 ± 0.01	0	6.58 ± 0.04	3
KTM125	SDW	8.58 ± 0.02	5.98 ± 0.02	0	0	0	0	0	0	0	0	0	0	4.20 ± 0.01	5.93 ± 0.02	2
KTM209		8.55 ± 0.03	7.33 ± 0.09	4.60 ± 0.21	4.16 ± 0.01	0	4.15 ± 0.01	0	0	0	0	0	4.39 ± 0.36	0	5.41 ± 0.18	5
KTM245		7.72 ± 0.03	5.00 ± 0.17	0	0	0	0	0	0	0	0	0	0	0	0	1
KTM294		8.05 ± 0.02	7.07 ± 0.08	5.42 ± 0.06	0	6.18 ± 0.06	0	0	4.54 ± 0.11	4.20 ± 0.01	5.01 ± 0.10	0	0	0	6.40 ± 0.08	6
KTM306		8.40 ± 0.03	6.98 ± 0.03	4.72 ± 0.14	4.18 ± 0.00	4.40 ± 0.32	4.31 ± 0.15	0	4.29 ± 0.21	0	0	0	0	0	6.62 ± 0.04	6
KTM342		8.07 ± 0.01	5.66 ± 0.10	4.98 ± 0.11	4.66 ± 0.14	4.69 ± 0.26	0	4.50 ± 0.20	4.42 ± 0.33	0	0	0	4.18 ± 0.01	0	5.46 ± 0.02	7
KTM362		8.61 ± 0.01	6.81 ± 0.02	4.46 ± 0.24	0	4.38 ± 0.22	0	4.55 ± 0.32	0	0	0	0	0	0	6.53 ± 0.09	4
KTM368		8.65 ± 0.02	6.89 ± 0.04	5.29 ± 0.09	4.56 ± 0.14	5.11 ± 0.12	4.28 ± 0.17	4.28 ± 0.17	4.17 ± 0.00	0	0	4.23 ± 0.01	0	0	6.71 ± 0.02	8
KTM412		8.68 ± 0.01	7.66 ± 0.01	6.58 ± 0.05	4.18 ± 0.02	6.70 ± 0.03	4.17 ± 0.00	0	0	0	0	4.16 ± 0.01	0	0	7.45 ± 0.02	6
KTM423		8.74 ± 0.06	6.43 ± 0.02	5.07 ± 0.35	4.69 ± 0.08	0	4.47 ± 0.02	4.43 ± 0.08	4.18 ± 0.02	4.14 ± 0.01	0	0	0	0	5.43 ± 0.13	7
KTM432		7.98 ± 0.02	5.42 ± 0.18	4.19 ± 0.01	4.17 ± 0.02	0	0	0	0	0	0	0	0	0	4.87 ± 0.27	3
KTM433	STW	7.58 ± 0.02	5.97 ± 0.10	4.32 ± 0.22	4.15 ± 0.01	0	4.16 ± 0.00	0	0	0	0	0	0	0	5.75 ± 0.08	4
KTM434		6.74 ± 0.01	5.75 ± 0.08	0	0	0	0	0	0	0	0	0	0	0	4.32 ± 0.21	1
KTM459		8.78 ± 0.03	6.21 ± 0.02	0	4.41 ± 0.17	4.68 ± 0.07	0	4.53 ± 0.22	0	0	4.20 ± 0.02	0	0	0	5.77 ± 0.14	5
KTM205	Spring	7.68 ± 0.03	5.24 ± 0.24	0	0	0	0	4.40 ± 0.30	0	0	0	0	0	0	0	2
KTM329		6.37 ± 0.09	0	0	0	0	0	0	0	0	0	0	0	0	0	0
KTM371		7.48 ± 0.03	5.15 ± 0.13	0	0	0	0	4.61 ± 0.11	0	0	0	0	0	0	4.75 ± 0.14	2
KTM446		7.51 ± 0.04	4.17 ± 0.01	0	0	4.16 ± 0.02	0	0	0	0	0	0	0	0	4.18 ± 0.02	2
KTM119	SS	6.69 ± 0.03	4.59 ± 0.11	0	0	4.19 ± 0.01	0	0	0	0	0	0	0	0	4.20 ± 0.02	2
KTM120		6.02 ± 0.02	4.44 ± 0.34	0	0	0	0	0	0	0	0	0	4.19 ± 0.01	0	0	2
KTM150		8.02 ± 0.03	7.18 ± 0.13	5.61 ± 0.02	4.57 ± 0.30	4.40 ± 0.34	0	5.02 ± 0.38	4.87 ± 0.21	0	0	0	0	0	6.62 ± 0.04	6
KTM159		5.49 ± 0.18	0	0	0	0	0	4.31 ± 0.19	0	0	0	0	0	0	4.30 ± 0.16	1
KTM392		7.38 ± 0.02	4.57 ± 0.36	0	0	0	0	0	0	0	0	0	0	0	0	1
KTM407		4.81 ± 0.30	4.51 ± 0.58	0	0	0	0	4.18 ± 0.02	0	0	0	0	0	0	0	2

**FIGURE 1:**
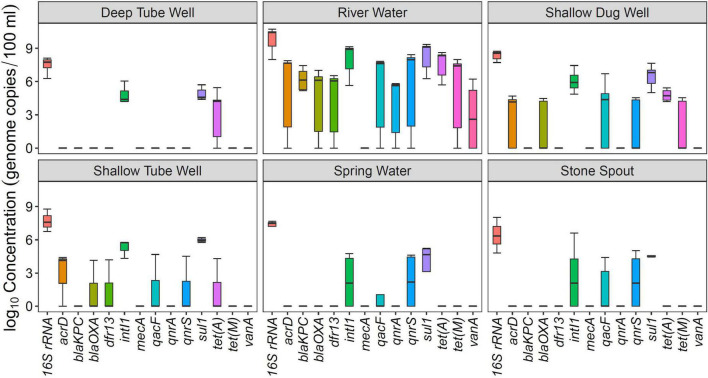
Concentration of antibiotic resistance genes (ARGs) in different drinking and environmental water sources.

Among the analyzed ARGs, *sul1* was detected in 94% (34/36) of the samples. The other ARG detected in more than 50% of the samples was *tet*(A) (Table 2). *intI1* was detected in 83% (30/36) of the samples. Absolute ARG concentrations were highest in river water samples, followed by shallow dug well and shallow tube well water samples (Figure 1).

**TABLE 2:** Positive ratios of antibiotic resistance genes (ARGs) and *intI1* in different drinking and environmental water samples.

Sample type	*sul1*	*tet*(A)	*acrD*	*qnrS*	*qacF*	*bla*_*OXA*_	*tet*(M)	*bla*_*KPC*_	*dfr13*	*qnrA*	*vanA*	*mecA*	*intI1*
Deep tube wells (*n* = 6)	6	4	1	1	0	1	0	1	0	0	0	0	6
Rivers (*n* = 6)	6	6	4	4	4	4	4	5	4	4	3	0	6
Shallow dug wells (*n* = 11)	11	9	7	4	6	5	5	2	1	2	2	1	10
Shallow tube wells (*n* = 3)	3	1	2	1	1	1	0	0	1	0	0	0	3
Springs (*n* = 4)	3	0	0	2	1	0	0	0	0	0	0	0	2
Stone spouts (*n* = 6)	5	1	1	3	2	0	1	0	0	0	1	0	3

Total (*n* = 36)	34	21	15	15	14	11	10	8	6	6	6	1	30
Positive ratio (%)	94	58	42	42	39	31	28	22	17	17	17	3	83

All river water samples contained *sul1, intI1*, and *tet*(A). Two river water samples were positive for all the tested ARGs, except for the *mecA* gene, and two other river water samples were positive for all the tested ARGs, except for *mecA* and *vanA* genes. Water samples collected at Budhanilkantha, the upstream site of the Dhobikhola River, and from the Godavari River contained only four ARGs where *bla*_*KPC*_ and *vanA* were detected in addition to the common ARGs, respectively. The sample collected from the Bishnumati River at Shovabhagavati Bridge had the highest concentration for all the ARGs, except for the *qnrA* gene. Absolute concentrations of 16S rRNA gene and other ARGs were usually 1–2 log_10_ higher in the river water samples containing 10 or 11 ARGs compared with samples containing four ARGs.

*sul1* was detected in all shallow dug well samples, while most of the samples were positive for *intI1* (10/11) and *tet*(A) (9/11). One sample contained 8 out of the 12 ARGs quantified in this study (KTM 368). *dfr13* was detected in only one sample, and *bla*_*KPC*_, *qnrA*, and *vanA* were detected only in two out of 11 samples. Maximum concentrations of *sul1* and *tet*(A) were 7.66 and 6.58 log_10_ gene copies/100 ml, respectively, while *intI1* concentration was 7.45 log_10_ gene copies/100 ml. Concentrations of other ARGs were at least 2 log_10_ lower than those of *sul1* and *tet*(A). Samples taken from the same shallow well 5 months apart showed a different ARG prevalence pattern. While the sample collected in February 2016 contained *vanA, acrD*, and *bla*_*OXA*_ in addition to *sul1* and *tet*(A), the sample collected in August 2016 was positive for *qacF, qnrA*, and *tet*(M) but not for *vanA* gene.

*sul1* and *intI1* were detected in all the shallow tube well water samples. In addition, *acrD, bla*_*OXA*_, *dfr13, qacF*, and *tet*(A) were sporadically detected. Concentrations of *sul1* and *intI1* were between 4.3 log_10_ and 6.2 log_10_ gene copies/100 ml. Other ARG concentrations were in the 4 log_10_ gene copies/100 ml range (Figure 1 and Table 1).

All deep tube well water samples were positive for *sul1, tet*(A), and *intI1*. *acrD, bla*_*KPC*_, *bla*_*OXA*_, and *qnrS* were sporadically detected in some samples, and other genes were not detected. The maximum number of ARGs detected in a deep tube well sample was three (Figure 1 and Table 1). Deep tube well samples had the lowest concentration of *sul1* gene among all sample types, which varied between 4.38 and 5.71 log_10_ gene copies/100 ml. It was 2 to 5 log_10_ lower than the *sul1* concentration in river water samples. *intI1* concentration also followed a similar trend (Table 1). Other detected ARGs were present in concentrations between 4.18 and 4.52 log_10_ gene copies/100 ml.

Compared with river water and shallow dug well water samples, fewer types of ARGs were detected from spring water samples. One spring water sample did not contain any of the measured ARGs, and *sul1* was the only positive ARG for the three remaining samples. *qacF* (1/3) and *qnrS* (2/3) were the other ARGs detected. *intI1* was detected in two samples. *sul1* was present at the highest concentration of 5.24 log_10_ gene copies/100 ml, while other ARGs and *intI1* concentrations were between 4 and 5 log_10_ gene copies/100 ml (Figure 1 and Table 1).

Similar to spring water samples, fewer types of ARGs were detected in stone spout samples. The highest number of ARGs detected in one sample was 6 (KTM150), while the other samples were positive for 1 or 2 ARGs. *sul1* was the predominant ARG (5/6), and the highest absolute concentration of *sul1* was 7.18 log_10_ gene copies/100 ml. In addition, *acrD, qacF, qnrS, tet*(A), *tet*(M), and *vanA* were detected in several samples with concentrations ranging between 4.19 and 5.61 log_10_ gene copies/100 ml. *intI1* was detected in 3 samples, and the concentration varied between 4.20 and 6.62 log_10_ gene copies/100 ml (Figure 1 and Table 1).

**FIGURE 2:**
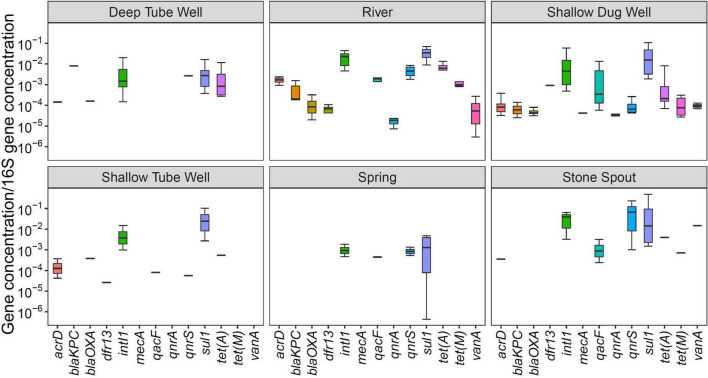
Relative abundance of antibiotic resistance genes (ARGs) in different drinking and environmental water sources.

In general, the relative abundance of *sul1* and *intI1* was high for all water source types. The relative abundance of *qnrS* gene was comparable with *sul1* and *intI1* in river and stone spout samples (Figure 2). *qacF* gene has shown higher relative abundance in exposed water sources such as river water, shallow dug wells, and stone spouts. The relative abundance of *sul1, intI1, vanA*, and *qnrS* genes in stone spout samples was high since the 16S rRNA concentration was lower than other water sources (Figure 2).

**FIGURE 3:**
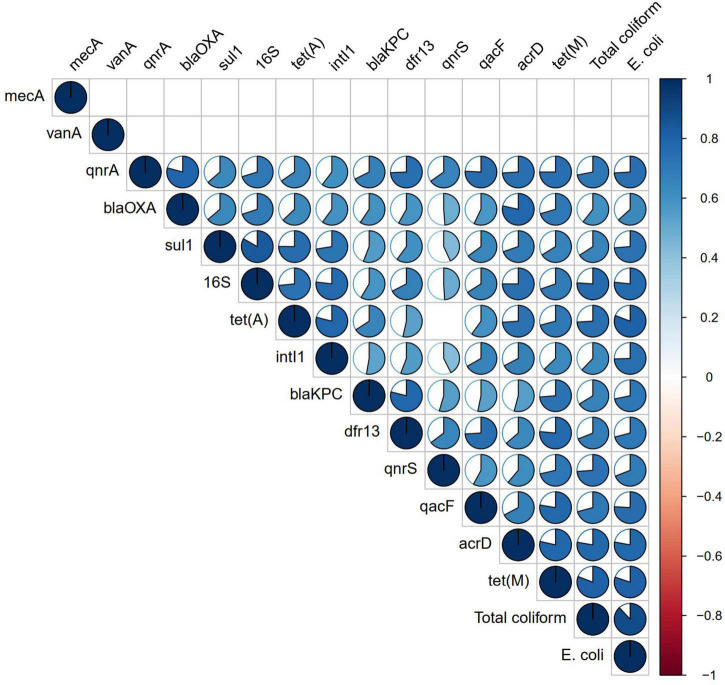
Correlation analysis of antibiotic resistance genes (ARGs) and fecal indicator bacteria. Correlation coefficient is shown in the pie, while non-significant correlations (*p* > 0.01) are kept blank.

**FIGURE 4:**
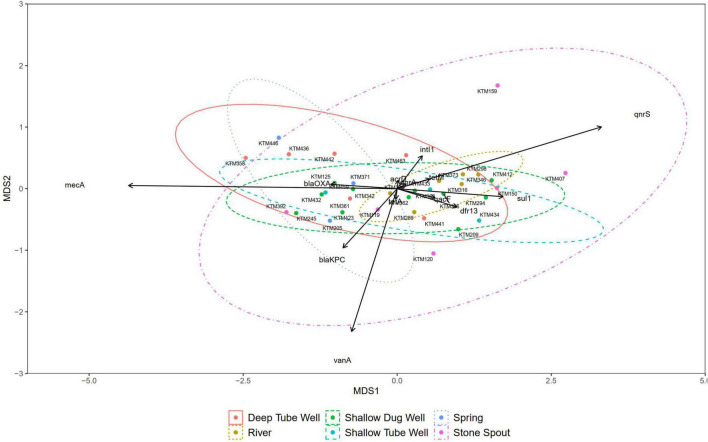
Non-metric multidimensional scaling (NMDS) plot of samples grouped by source type (stress = 0.088).

Correlation analysis of ARGs showed moderate to very high correlations (*r* > 0.5, *p* < 0.01) among each other, with total coliforms and *E. coli* except for *vanA, qnrS*, and *mecA* (Figure 3). According to the NMDS analysis, river water samples are ordinated close together as expected, and samples containing a high number of measured ARGs are sub-clustered within the group. Stone spout samples are ordinated far apart confirming the dissimilarities in the ARGs present in each sample (Figure 4).

## Discussion

The ARG concentrations in drinking water and environmental water sources collected from the Kathmandu Valley were quantified using ddPCR. Among the surface water and groundwater samples analyzed, at least one ARG was detected in 35 (97%) out of 36 samples. Maximum concentrations of different ARGs varied between 4.2 (*mecA*) and 9.34 (*sul1*) log_10_ gene copies/100 ml.

River water samples contained the highest concentration of all ARGs, except for the *mecA* gene. A recent study reported similar results where ARG concentrations in the samples from the mid-stream and downstream of the Bagmati River were 8–9 log_10_ copies/100 ml. *dfr13* gene associated with cotrimoxazole resistance and detected in hospital isolates (Acharya and Wilson, 2019) was detected in all four highly contaminated river water samples. In Nepal, 74% of the hospitals do not have wastewater treatment facilities (Joshi et al., 2017). Direct discharge of untreated municipal and hospital wastewaters and improperly treated wastewater to rivers in highly populated areas were the possible reasons for this observation (Acharya and Wilson, 2019; Thakali et al., 2020, 2021). A low level of ARG contamination at KTM289, the upstream site of the Bagmati River, and KTM374, the upstream site of the Godavari River, with low population density corroborate this. Since river water is being extensively used for irrigation purposes, some of these ARGs may end up in fresh produce and possibly pose a human health risk (Amarasiri et al., 2020; Sorinolu et al., 2021).

Spring water and stone spout samples contained the fewest ARGs. The water in the stone spouts flows continuously in underground channels (Tripathi et al., 2018) and is less exposed to contaminants, which may have been reflected as few types of detected ARGs.

In addition to *sul1* and *tet*(A), *acrD* was the most prevalent ARG in shallow dug wells. Studies on the animal samples, such as milk and poultry carcasses, have shown that bacterial isolates from such samples are resistant to aminoglycosides, such as gentamicin. Moreover, gentamycin is being used to treat mastitis diseases in cattle (Central Veterinary Laboratory, 2021). A number of meat shops, hospitals, and a pharmacy were present in the surroundings (within 200 m) of the shallow wells where 8 ARGs were detected. Contamination of shallow dug wells by wastewater from such establishments and discharging of untreated wastewater and partially treated municipal, industrial, and hospital wastewaters to rivers may have affected shallow groundwater (Mackie et al., 2006; Elliott et al., 2018; Bajracharya et al., 2020; Hubbard et al., 2020).

*qacF* disinfectant resistant gene was detected in all water source types, except for deep tube well samples. Quaternary ammonium compounds are ubiquitous in soaps and sanitizers. Washing activities conducted in rivers and in the vicinity of shallow wells may have acted as a selection pressure that contributed to the development of antibiotic-resistant bacterial strains (Allen and Canadian Paediatric Society, 2006; Zou et al., 2014).

The results of this study highlight that ARGs are prevalent in surface and groundwater samples that are used for drinking, bathing, and other household activities. A recent critical review highlighted the suitability of *sul1, intI1, tet*(A), *vanA*, and *bla*_*CTX–M*_ as potential ARG targets to standardize the ARG monitoring in surface water and wastewater (Keenum et al., 2022). In this study, *sul1, intI1, tet*(A), and *vanA* were quantified not only in surface water but also in groundwater samples. The results complement the selection of the above genes and further suggest their suitability for monitoring the ARGs in groundwater.

*sul1* and *intI1* were present in 94 and 83% of samples analyzed in this study, respectively, implying anthropogenic contamination (Pruden et al., 2012; Gillings et al., 2015). Maximum concentrations of those genes were also 1–2 log_10_ higher than those of other ARGs. It is possible to speculate a similar situation in other LMICs (Devarajan et al., 2016; Rafraf et al., 2016) where antibiotic usage-related regulations are weak (Nadimpalli et al., 2018; Larsson and Flach, 2021). Under such conditions, ARGs such as *sul1* and *intI1* may be ubiquitous in aquatic environments and therefore may be only used to evaluate background conditions. Moreover, deep tube well water samples were positive only for *sul1, tet*(A), and *intI1*. It may suggest that these ARGs were already being present in those water sources for a long time rather than being a recent anthropogenic input. It also strengthens the suggestion of using those genes only as background ARG contamination in LMICs. However, clinically relevant ARGs, such as *bla*_*KPC*_, *bla*_*OXA*_, *qnrS*, and *vanA*, which are not frequently detected in environmental settings were sporadically detected in samples from almost all the source water types (Table 2). Clinical *intI1* has shown to better correlate with ARGs (Gillings et al., 2015; Zheng et al., 2020). High concentration and relative abundance of disinfectant resistance gene, *qac*F, in rivers and shallow dug wells may also be representative of the anthropogenic contamination (Szekeres et al., 2018). Therefore, monitoring of such ARGs may provide valuable information on the ARGs contamination status of aquatic environments in LMICs, including Nepal.

The NMDS did not show any dissimilarities between most of the source water types, suggesting the cross-contamination or presence of a common contamination origin. However, a similar gene clustering pattern was not observed in stone spout water samples. Therefore, ARGs detected in different stone spout water samples may reflect the ARGs present in their respective origins. Further studies are necessary to identify the origins of antibiotic resistance in aquatic environments of the Kathmandu Valley.

The highest ARG concentration detected in drinking water source samples varied between 4.18 and 7.66 log_10_ copies/100 ml for different ARGs. Ding et al. (2020) gavaged extracellular RK2 plasmid containing apramycin resistance gene at around 10^11^ copies/ml concentration into mice and observed the bacterial transformation occurring in the proximal and distal jejuna and ilea for 2 days. After 2 days, transformants acted as the plasmid donors, and the RK2 plasmid was disseminated by conjugation for another 6 days before reaching a stable concentration (Ding et al., 2020). The same scenario might occur in humans exposed to ARGs in drinking water which poses a human health risk.

The highest ARG concentrations detected in river water samples varied between 5.82 and 9.34 log_10_ copies/100 ml for different ARGs. Wastewater treatment plant effluents in the Kathmandu Valley were shown to contain ARG concentrations between 10^8^ and 10^11^ genome copies/L (Thakali et al., 2021). Those discharges may have been a major reason for the high concentrations of ARGs in river water. Long-term irrigation with treated wastewater increased the relative abundance of *sul1* and *intI1* in the groundwater microbiome (Kampouris et al., 2022) and *intI1* concentration in dry soil (Zammit et al., 2020). A similar phenomenon can be expected in the Kathmandu Valley, where river water containing high concentrations of ARGs was used for agriculture. Even though the ARG loads in produce, such as tomatoes and beans, are 0.1–0.01% of the concentrations in dry soil (Cerqueira et al., 2019), it may still pose a human health risk when consuming raw products.

Total coliforms and *E. coli* are used as indicators for fecal contamination. In this study, total coliforms and *E. coli* were detected at the highest concentrations of 5.72 and 4.51 log_10_ MPN/100 ml in drinking water sources, respectively, which is 2–3 log_10_ lower than those in river water. Uprety et al. (2020) collected 2,822 water samples from 37 districts out of 77 in Nepal including source water, communal tap water, reservoir water, and household stored water and analyzed for the presence of *E. coli*. The mean *E. coli* concentration was 70 ± 550 CFU/100 ml. Assuming 8% of the *E. coli* is pathogenic in 2 L of daily water consumption, 4 × 10^–2^ disability adjusted life year loss (DALY Loss) per person per year (pppy) was calculated (Uprety et al., 2020). Calculated DALY loss pppy exceeds 10^–4^ DALYpppy which was considered an adequate margin of public-health safety related to waterborne diarrheal diseases (Mara, 2011). Even though MPN-based *E. coli* calculations are shown to be higher than the culture-based estimations (Prats et al., 2008), it is possible to expect that the concentration of *E. coli* detected in our study may also exceed the acceptable DALYpppy loss for waterborne diseases. If horizontal gene transfer occurs and the antibiotic-resistant *E. coli* concentrations were increased, it may significantly increase the DALYpppy loss.

In the ddPCR quantification method used in this study, the limit of quantification was around 1.2 genome copies/reaction, which is comparable to a previous study (Cavé et al., 2016). A previous study by our group which quantified ARGs in drinking water samples from Kathmandu Valley using the qPCR technique reported the limit of quantification as 5 copies/reaction which may get influenced by the amplification efficiency and the inhibitory substances (Thakali et al., 2022). Therefore, ddPCR can be suggested as a sensitive and specific quantitative analysis method usable in routine monitoring studies for ARGs in aquatic environments even when the monitoring targets are available in very low concentrations or inhibitory substances are present (Ishii, 2020).

In this study, grab sampling was conducted, and the ARG concentrations in each sample were quantified. One limitation of the grab sampling is the inability to evaluate the variations of ARG concentrations within each day. It might affect the ARG load in river water, depending on the wastewater treatment plant and hospital wastewater discharge schedules. It may not directly affect the ARG concentrations in groundwater sources unless there are anthropogenic activities in the vicinity of the unimproved shallow dug wells. As the ARG concentrations in river systems have shown seasonal variations (Harnisz et al., 2020), further studies are necessary to clarify the phenomenon in the river water of Nepal.

This study highlights the ubiquitous presence of ARGs in drinking and environmental water sources of the Kathmandu Valley. ARGs in aquatic environments have the potential to pose a human health risk if they are being transferred to human pathogens (Wright, 2010; Tomova et al., 2015). As the improvement of basic sanitation can reduce antibiotic resistance dissemination (Graham et al., 2019), interventions associated with sanitation may contribute to minimizing the ARG loads in aquatic environments. Affordable wastewater treatment systems and stringent regulations may also play a role in reducing antibiotic resistance dissemination and potential human health risks.

## Data availability statement

The original contributions presented in this study are included in the article/Supplementary material, further inquiries can be directed to the corresponding author.

## Author contributions

EH, JS, and KS initiated this project. MA, TF, EH, and KS contributed to the design of the experiment. BM processed the samples. TT, BM, and MA conducted the experiments and did data processing and analysis. MA, BM, TF, JS, EH, and KS wrote and edited the manuscript. All authors approved the manuscript for publication.
