# Supplementary material for: Prevalence of antibiotic resistance genes in drinking and environmental water sources of the Kathmandu Valley, Nepal
Source: Front Microbiol. 2022 Aug 22;13:894014. doi: 10.3389/fmicb.2022.894014 (PMC9441849; doi:10.3389/fmicb.2022.894014)
Supplement: Supplementary file 1 [file Data_Sheet_1.PDF]

Supplementary Material

Prevalence of Antibiotic Resistance Genes (ARGs) in Drinking and Environmental Water Sources of the Kathmandu Valley, Nepal

Mohan Amarasiri[1,*], Tsubasa Takezawa[1], Bikash Malla[2], Takashi Furukawa[1], Jeevan B Sherchand[3], Eiji Haramoto [2], Kazunari Sei[1]

[1]Laboratory of Environmental Hygiene, School of Allied Health Sciences, Kitasato University, Sagamihara-Minami, Kanagawa, 252-0373, Japan.

[2]Interdisciplinary Center for River Basin Environment, University of Yamanashi, 4-3-11 Takeda, Kofu 400-8511, Japan.

[3]Institute of Medicine, Tribhuvan University Teaching Hospital, Kathmandu, Nepal.

*** Correspondence:** mohan@kitasato-u.ac.jp

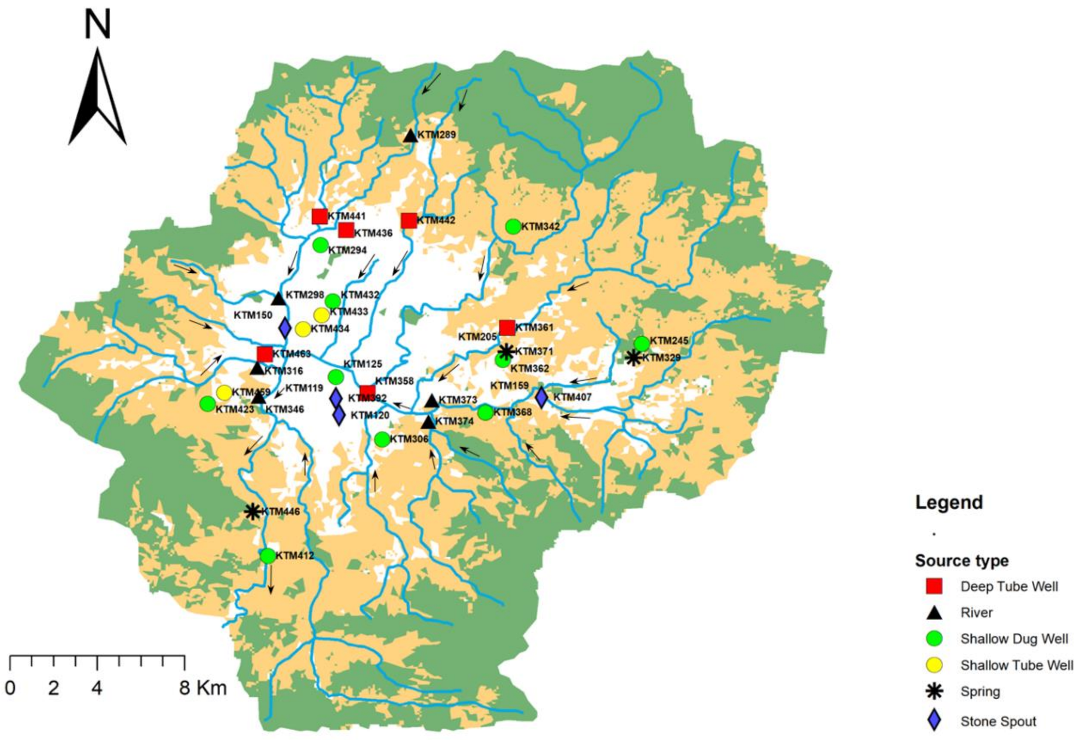

Supplementary Figure 1. Sampling locations of this study. Arrows indicate the river flow direction.

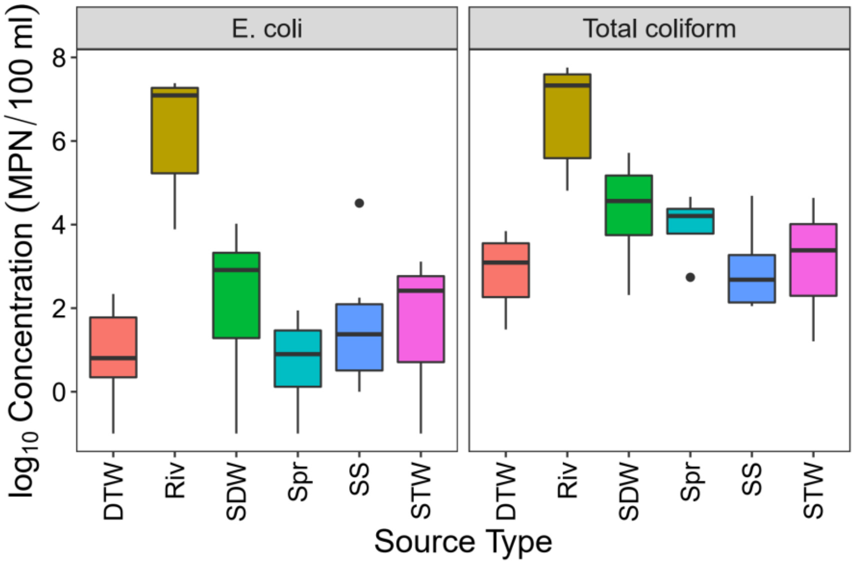

Supplementary Figure 2. Concentrations of *E. coli* and total coliforms in the water samples collected from different water sources. DTW: Deep Tube Well, Riv: River Water, SDW: Shallow Dug Well, Spr: Spring, SS: Stone Spout, and STW: Shallow Tube Well

Supplementary Table 1. Primer pairs and positive controls used in quantifying ARGs from water samples

	Gene	Antibiotics class of resistance		Primers and Standards	Amplicon Length (bp)	Reference
1	*16S rRNA*		357F	CTCCTACGGGAGGCAGCAG	200	(Turner et al., 1999)
			556R	CTTTACGCCCAGTAATTCCG		(Demarta et al., 1999)
			PC	GGAACTGAGACACGGTCCAGACTCCTACGGGAGGCAGCAGTGGGGAATATTGCA<br>CAATGGGCGCAAGCCTGATGCAGCCATGTCGCGTGTATGAAGAAGGCCTTCGGG<br>TTGTAAAGTACTTTCAGCGGGGAGGAAGGGAGTAAAGTTAATACCTTTGCTCATT<br>GACGTTACCCGCAGAAGAAGCACCGGCTAACTCCGTGCCAGCAGCCGCGGTAAT<br>ACGGAGGGTGCAAGCGTTAATCGGAATTACTGGGCGTAAAGCGCACGCAGGCGG<br>TTTGTTAAGTCAGATGTGAAATCCCCGGGCTCAACCTGGGAACTGCATCTGATAC<br>TGGCAAGCTTGAGTCTCGTAGAGGGGGGTAGAATTCCAGGTGTAGCGGTGAAAT<br>GCGTAGAGATCTGGAGGAATACCGGTGGCGAAGGCGGCCCCCTGGACGAAGACT<br>GACGCTCAGGTGCGAAAGCGTGGGGAGCAAACAGGATTAGATACCCTGGTAGTC<br>CACGCCGTAAACGATGTCGACTTGGAGGTTGTGCCCTTGAGGCGTGGCTTCCGGA<br>GCTAACGCGTTAAGTCGACCGCCTGGGGAGTACGGCCGCAAGGTTAAAACTCAA<br>ATGAATTGACGGGGGCCCGCACAAGCGGTGGAGCA		This study
2	*acrD*	Multidrug efflux pumps, Aminoglycosides	F	GGCAATCCTGTTGTGTCTGA	145	
			R	ACATGAGATTATCGAGGCCG		
			PC	CCTGGGTGCTGGCAATCCTGTTGTGTCTGACAGGGACCCTGGCGATTTTTTCATTG<br>CCCGTTGAACAATACCCCGATCTCGCGCCACCGAATGTGCGAGTGACCGCTAACT<br>ATCCCGGCGCATCGGCCCAGACGCTGGAAAACACCGTGACCCAGGTTATCGAGC<br>AAAATATGACCGGCCTCGATAATCTCATGTATATGTCATC		(Sandberg et al., 2018)
3	*bla*KPC	β-lactams	F	GATACCACGTTCCGTCTGG	213	
			R	GCAGGTTCCGGTTTTGTCTC		
			PC	CTCTATCGGCGATACCACGTTCCGTCTGGACCGCTGGGAGCTGGAGCTGGAGCTG<br>AACTCCGCCATCCCAGGCGATGCGCGCGATACCTCATCGCCGCGCGCCGTGACGG<br>AAAGCTTACAAAAACTGACACTGGGCTCTGCACTGGCTGCGCCGCAGCGGCAGC<br>AGTTTGTTGATTGGCTAAAGGGAAACACGACCGGCAACCACCGCATCCGCGCGG<br>CGGTGCCGGCAGACTGGGCAGTCGGAGACAAAACCGGAACCTGCGGAGTGTATG		(Sandberg et al., 2018)

4	*bla*OXA-48	β-lactams	F	TGTTTTTGGTGGCATCGAT	177	(Monteiro et al., 2012)
			R	GTAAMRATGCTTGGTTCGC		
			PC	TGCGTGTATTAGCCTTATCGGCTGTGTTTTTGGTGGCATCGATTATCGGAATGCCT<br>GCGGTAGCAAAGGAATGGCAAGAAAACAAAAGTTGGAATGCTCACTTTACTGAA<br>CATAAATCACAGGGCGTAGTTGTGCTCTGGAATGAGAATAAGCAGCAAGGATTT<br>ACCAATAATCTTAAACGGGCGAACCAAGCATTTTTACCCGCATCTA		This study
5	*dfr13*	Cotrimoxazole	F	AATCGGTCCGCATTTATCTG	134	
			R	TTGGTAAGGGCTTGCCTATG		(Sandberg et al., 2018)
			PC	ATGAACCCGGAATCGGTCCGCATTTATCTGGTCGCTGCCATGGGTGCCAATCGGG<br>TTATTGGCAATGGTCCCGATATCCCCTGGAAAATCCCAGGTGAGCAGAAGATTTT<br>TCGCAGGCTCACCGAGAGCAAAGTGGTCGTTATGGGCCGCAAGACATTTGAGTC<br>CATAGGCAAGCCCTTACCAAACCGCCACAC		
6	*IntI1*		F	CCTCCCGCACGATGATC	246	
			R	TCCACGCATCGTCAGGC		(Sandberg et al., 2018)
			PC	GAGCCCTTGCCCTCCCGCACGATGATCGTGCCGTGATCGAAATCCAGATCCTTGA<br>CCCGCAGTTGCAAACCCTCACTGATCCGCATGCCCGTTCCATACAGAAGCTGGGC<br>GAACAAACGATGCTCGCCTTCCAGAAAACCGAGGATGCGAACCACTTCATCCGG<br>GGTCAGCACCACCGGCAAGCGCCGCGACGGCCGAGGTCTTCCGATCTCCTGAAG<br>CCAGGGCAGATCCGTGCACAGCACCTTGCCGTAGAAGAACAGCAAGGCCGCCAA<br>TGCCTGACGATGCGTGGAGACCGAAACC		
7	*mecA*	Methicillin	F	GCAATCGCTAAAGAACTAAG	222	(Fang and Hedin, 2003)
			R	GGGACCAACATAACCTAATA		
			PC	AAGATTATAAAGCAATCGCTAAAGAACTAAGTATTTCTGAAGACTATATCAAAC<br>AACAAATGGATCAAAATTGGGTACAAGATGATACCTTCGTTCCACTTAAAACCGT<br>TAAAAAAATGGATGAATATTTAAGTGATTTCGCAAAAAAATTTCATCTTACAACT<br>AATGAAACAAAAAGTCGTAACTATCCTCTAGAAAAAGCGACTTCACATCTATTAG<br>GTTATGTTGGTCCCATTAACTCTGAAG		This study
8	*qacF*		F	TGGCTGTTTCAATCTTTGGC	132	(Sandberg et al., 2018)

		Quaternary ammonium compounds	R	GCCCATACAGCGTAAGCAAT		
			PC	TGGATATTTCTGGCTGTTTCAATCTTTGGCGAGGTCATCGCAACTTCCGCACTGAA GTCTAGCCATGGATTCACTAGGTTAGTTCCTTCCGTTGTAGTTGTGGCTGGTTACG GGCTTGCGTTCTATTTCTTGTCTCTCGCGCTCAAGTCCATTCCGGTCGGTATTGCT TACGCTGTATGGGCTGGGCTTGGC		
9	*qnrA*	Fluoroquinolones	F	AGGATTTCTCACGCCAGGATT	83	(Sandberg et al., 2018)
			R	CCGCTTTCAATGAAACTGCA		
			PC	TTTCAGCAAGAGGATTTCTCACGCCAGGATTTGAGTGACAGCCGTTTTCGCCGCT GCCGCTTTTATCAGTGTGACTTCAGCCATTGCCAGCTAAGGGATGCCAGTTTCGA GGATTGCAGTTTCATTGAAAGCGGCGCCATCGAA		
10	*qnrS*	Fluoroquinolones	F	GTGAGTAATCGTATGTACTTTTGC	169	(Guillard et al., 2011)
			R	AAACACCTCGACTTAAGTCT		
			PC	TTCGTCAACTGCAAGTTCATTGAACAGGGTGATATCGAAGGCTGCCACTTTGATG TCGCAGATCTTCGTGATGCAAGTTTCCAACAATGCCAACTTGCGATGGCAAACTT CAGTAATGCCAATTGCTACGGTATAGAGTTCCGTGCGTGTGATTTAAAAGGTGCC AACTTTTCCCGAACAAACTTTGCCCATCAAGTGAGTAATCGTATGTACTTTTGCTC AGCATTTATTTCTGGATGTAATCTTTCCTATGCCAATATGGAGAGGGTTTGTTTAG AAAAATGTGAGTTGTTTGAAAATCGCTGGATAGGAACGAACCTAGCGGGTGCAT CACTGAAAGAGTCAGACTTAAGTCGAGGTGTTTTTTCCGAAGATGTCTGGGGGCA ATTTAGCCTACAGGGTGCCAATTTATGCCACGCCGAACTCGACGGTTTAGATCCC CGCAAA		This study
11	*sul1*	Sulfonamides	F	CCGTTGGCCTTCCTGTAAAG	29	(Sandberg et al., 2018)
			R	TTGCCGATCGCGTGAAGT		
			PC	GGTGTCGCGGAAATCCTTCTTGGGCGCCACCGTTGGCCTTCCTGTAAAGGATCTG GGTCCAGCGAGCCTTGCGGCGGAACTTCACGCGATCGGCAATGGCGCTGACTAC GTCCGCACCCACGCGCCTGGAGA		This study
12	*tet(A)*	Tetracycline	F	GCTACATCCTGCTTGCCTTC	170	(Sandberg et al., 2018)
			R	CATAGATCGCCGTGAAGAGG		

			PC	GACGGCACAGGCTACATCCTGCTTGCCTTCGCGACACGGGGATGGATGGCGTTCC<br>CGATCATGGTCCTGCTTGCTTCGGGTGGCATCGGAATGCCGGCGCTGCAAGCAAT<br>GTTGTCCAGGCAGGTGGATGAGGAACGTCAGGGGCAGCTGCAAGGCTCACTGGC<br>GGCGCTCACCAGCCTGACCTCGATCGTCGGACCCCTCCTCTTCACGGCGATCTAT<br>GCGGCTTCTAT		
13	*tet(M)*	Tetracycline	F	TTAGGAAGCGTGGACAAAGG	151	This study
			R	TTGTGGGAATCCCCATTTTC		
			PC	ATTAGGAAGCGTGGACAAAGGTACAACGAGGACGGATAATACGCTTTTAGAACG<br>TCAGAGAGGAATTACAATTCAGACAGGAATAACCTCTTTTCAGTGGGAAAATAC<br>GAAGGTGAACATCATAGACACGCCAGGACATATGGATTTCTTAGCAGAAGTATA<br>TCGTTCATTATCAGTTTTAGATGGGGCAATTCTACTGATTTCTGCAAAAGATGGC<br>GTACAAGCACAAACTCGTATATTATTTCATGCACTTAGGAAAATGGGGATTCCCA<br>CAATCTTTTTTATCAATAAGATTGACCAAAATGGAATTGATTTATCAACGGTTTAT<br>CAGGATATTAAAGAGAAACTTTCTGCCGAAATTGTAATCAAACAGAAGGTAGAA<br>CTGTATCCTAATATGTGTGTGACGAACTTTACCGAATCTGAACA		(Sandberg et al., 2018)
14	*vanA*	Glycopeptides	F	GTAGGCTGCGATATTCAAAGC	189	
			R	CGATTCAATTGCGTAGTCCAA		
			PC	TATCCCTTTTGTAGGCTGCGATATTCAAAGCTCAGCAATTTGTATGGACAAATCG<br>TTGACATACATCGTTGCGAAAAATGCTGGGATAGCTACTCCCGCCTTTTGGGTTA<br>TTAATAAAGATGATAGGCCGGTGGCAGCTACGTTTACCTATCCTGTTTTTGTTAA<br>GCCGGCGCGTTCAGGCTCATCCTTCGGTGTGAAAAAAGTCAATAGCGCGGACGA<br>ATTGGACTACGCAATTGAATCGGCAAGACAAT		(Sandberg et al., 2018)

References

Demarta, A., Tonolla, M., Caminada, A. P., Ruggeri, N., and Peduzzi, R. (1999). Signature region within the 16S rDNA sequences of Aeromonas popoffii. *FEMS Microbiol. Lett.* 172, 239–246. doi:10.1016/S0378-1097(99)00029-4.

Fang, H., and Hedin, G. (2003). Rapid screening and identification of Methicillin-Resistant Staphylococcus aureus from clinical samples by selective-broth and Real-Time PCR assay. *J. Clin. Microbiol.* 41, 2894–2899. doi:10.1128/JCM.41.7.2894-2899.2003.

Guillard, T., Moret, H., Brasme, L., Carlier, A., Vernet-Garnier, V., Cambau, E., et al. (2011). Rapid detection of qnr and qepA plasmid-mediated quinolone resistance genes using real-time PCR. *Diagn. Microbiol. Infect. Dis.* 70, 253–259. doi:10.1016/j.diagmicrobio.2011.01.004.

Monteiro, J., Widen, R. H., Pignatari, A. C. C., Kubasek, C., and Silbert, S. (2012). Rapid detection of carbapenemase genes by multiplex real-time PCR. *J. Antimicrob. Chemother.* 67, 906–909. doi:10.1093/jac/dkr563.

Sandberg, K. D., Ishii, S., and LaPara, T. M. (2018). A Microfluidic Quantitative Polymerase Chain Reaction Method for the Simultaneous Analysis of Dozens of Antibiotic Resistance and Heavy Metal Resistance Genes. *Environ. Sci. Technol. Lett.* 5, 20–25. doi:10.1021/acs.estlett.7b00552.

Turner, S., Pryer, K. M., Miao, V. P. W., and Palmer, J. D. (1999). Investigating deep phylogenetic relationships among cyanobacteria and plastids by small subunit rRNA sequence analysis. *J. Eukaryot. Microbiol.* 46, 327–338. doi:10.1111/j.1550-7408.1999.tb04612.x.
